# Paeoniflorin regulates osteoclastogenesis and osteoblastogenesis via manipulating NF-κB signaling pathway both *in vitro* and *in vivo*

**DOI:** 10.18632/oncotarget.23677

**Published:** 2017-12-27

**Authors:** Yanmao Wang, Jiezhi Dai, Yu Zhu, Wanrun Zhong, Shengdi Lu, Hua Chen, Yimin Chai

**Affiliations:** ^1^ Department of Orthopedic Surgery, Shanghai Jiao Tong University Affiliated Sixth People’s Hospital, Shanghai 200233, PR China

**Keywords:** paeoniflorin, osteoclastogenesis, osteoblastogenesis, NF-κB, OVX

## Abstract

The metabolic balance between synthesis and resorption of the bone is maintained by osteoblasts and osteoclasts, respectively. Identification of agents that stimulate bone formation and suppress excessive osteoclast formation, may aid in preventing and treating conditions like osteoporosis and periprosthetic loosening. Paeoniflorin is a natural product derived from *Paeonia lactiflora* Pall with anti-inflammatory, analgesic, and diuretic properties. However, the effect of paeoniflorin on osteoclastogenesis and osteoblastogenesis is unknown. Herein, we demonstrated that paeoniflorin has a dose-dependent suppressive action on RANKL-evoked osteoclast differentiation and bone resorption, achieved by inhibiting the NF-κB pathway and subunit p65 nuclear translocation. Simultaneously, paeoniflorin was also found to stimulate osteoblast differentiation and bone mineralization, in addition to rescuing TNFα-impaired osteoblastogenesis. At the molecular level, paeoniflorin was found to inhibit NF-κB transcriptional activity and stimulate osteoblastogenesis-related marker gene expression (ALP, osteocalcin, OPN and Runx2), a trend that was inhibited by p65 overexpression. In ovariectomized mice, paeoniflorin was found to improve osteoblast activity, inhibit osteoclast activity, and thus, reduce ovariectomy-induced osteoporosis. Our study demonstrated that paeoniflorin simultaneously suppressed osteoclastogenesis and facilitated osteoblastogenesis by manipulating the actions of NF-κB. Therefore, paeoniflorin may serve as an ideal therapeutic antidote for osteoporosis.

## INTRODUCTION

Osteoblast-regulated bone formation and osteoclast-controlled bone resorption comprise a reasonably balanced homeostatic system. Conditions such as osteoporosis may occur following defective osteoblastic-bone formation or excessive osteoclastic-bone resorption. Therefore, the treatment of osteoporosis requires agents that stimulate bone formation or inhibit bone resorption. Agents such as bisphosphonates, which inhibit bone resorption would eventually result in decreased bone formation as well. Conversely, a concomitant acceleration of bone resorption is observed with the use of anabolic agents like the parathyroid hormone (PTH). It appears that drugs acting on a single target may fail owing to the coupling effect of bone formation and resorption. Consequently, there is a search for new dual-action therapeutic agents for osteoporosis that simultaneously suppress bone resorption and accelerate bone formation [1, 2].

Paeoniflorin, a Chinese herbal medicine ingredient, is the principle component of the total glucosides of peony, which is isolated from the dried root of *Paeonia lactiflora* Pall (Family, Ranunculaceae). This agent is used in traditional Chinese prescriptions for its anti- inflammatory, analgesic, and diuretic effects [3]. In addition, paeoniflorin has been confirmed to have antiphlogistic, immunoregulatory, anti-allergic [4], antinociceptive [5], antioxidative [6], and antiproliferative [7] functions. Since 1998, the US Food and Drug Administration has permitted the sale of this drug. Particularly, prof. Li has recently demonstrated that paeoniflorin could alleviate atherosclerotic inflammation by inhibiting the TLR4/MyD88/ NF-κB pathway, and suggested that paeoniflorin may be a potential therapy for atherosclerosis [8]. In this present study we mainly focused on the influence of paeoniflorin on osteoclast-regulated bone resorption and BMSC (bone marrow stem cell)-regulated osteoblast differentiation, and also explained the possible molecular mechanisms of its actions.

The organic matrix of the bone is formed by osteoblasts (OBs). This matrix provides a platform for mineralization, which is important to establish and maintain bone structure. The various signaling factors that are involved in the differentiation process of OB include fibroblast growth factors, parathyroid hormone-related protein, bone morphogenic proteins (BMPs), transforming growth factor ß (TGF ß), Wnts, and members of the growth hormone/IGF family [9–11]. More recently, OB signaling was thought to be mainly regulated by NF-κB, which appeared to restrain OB differentiation [9, 12]. Preliminary observations that phlegmonosis and TNFα suppressed *in vitro* and *in vivo* bone formation suggested the relationship between NF-κB and OBs [13–15]. Other reports have identified that NF- κB intervenes in the activity of SMAD, which is located downstream of BMP2 or TGF-ß [16, 17]. Furthermore, Yamazaki *et al.* proposed that in the nucleus, p65 and Smad1-Smad5 complex act on each other. Further, p65 may interrupt its relation with the target promoter [18].

Osteoclasts (OCs) possess the ability to resorb bone, which is crucial in maintaining homeostasis, as well as in eliminating pathological bone tissue [19]. In the monocyte lineage, hematopoietic progenitors facilitate the formation of OCs. These progenitors rely on the M-CSF (CSF-1) for their survival. On coming in contact with RANKL (Receptor activator of nuclear factor kappa-B ligand), precursor cells differentiate to the OC lineage and upregulate c-fos and NFATc1. This differentiation requires two transcription factors [20, 21]. During this process, some OC precursors become polynuclear by fusion, while other mature OCs undergo polarization to construct a sealing zone. The entire process of differentiation requires the mandatory involvement of RANKL in forming the lineage and stimulating the mature cells to initiate resorption [22–24]. The activation of the signaling cascades is led by M-CSF and RANKL. The activation of NF-κB is the most important step, and in its absence there would not be any production of OCs [9, 25]. Other factors including inflammatory cytokines also strongly influence the differentiation of OC, mainly by stimulating the activation of NF-κB. Following the activation the NF-κB pathway, the pathway translocates to the nucleus with the release of subunit p65. This is accompanied by the expression of multiple osteoclastogenesis-related genes, which are stimulated by cathepsin K, c-Src, and tartrate-resistant acid phosphatase (TRAP) [23, 26, 27].

At present, there is insufficient research focus on the role of paeoniflorin on osteoblastogenesis and osteoclastogenesis, either *in vitro* or *in vivo*, or on the potential molecular mechanisms of paeoniflorin. This study aimed at analyzing the role of paeoniflorin on bone resorption and formation, and also attempted to describe the molecular basis of its action.

## RESULTS

### Influence of paeoniflorin on cell viability

**Figure 1:**
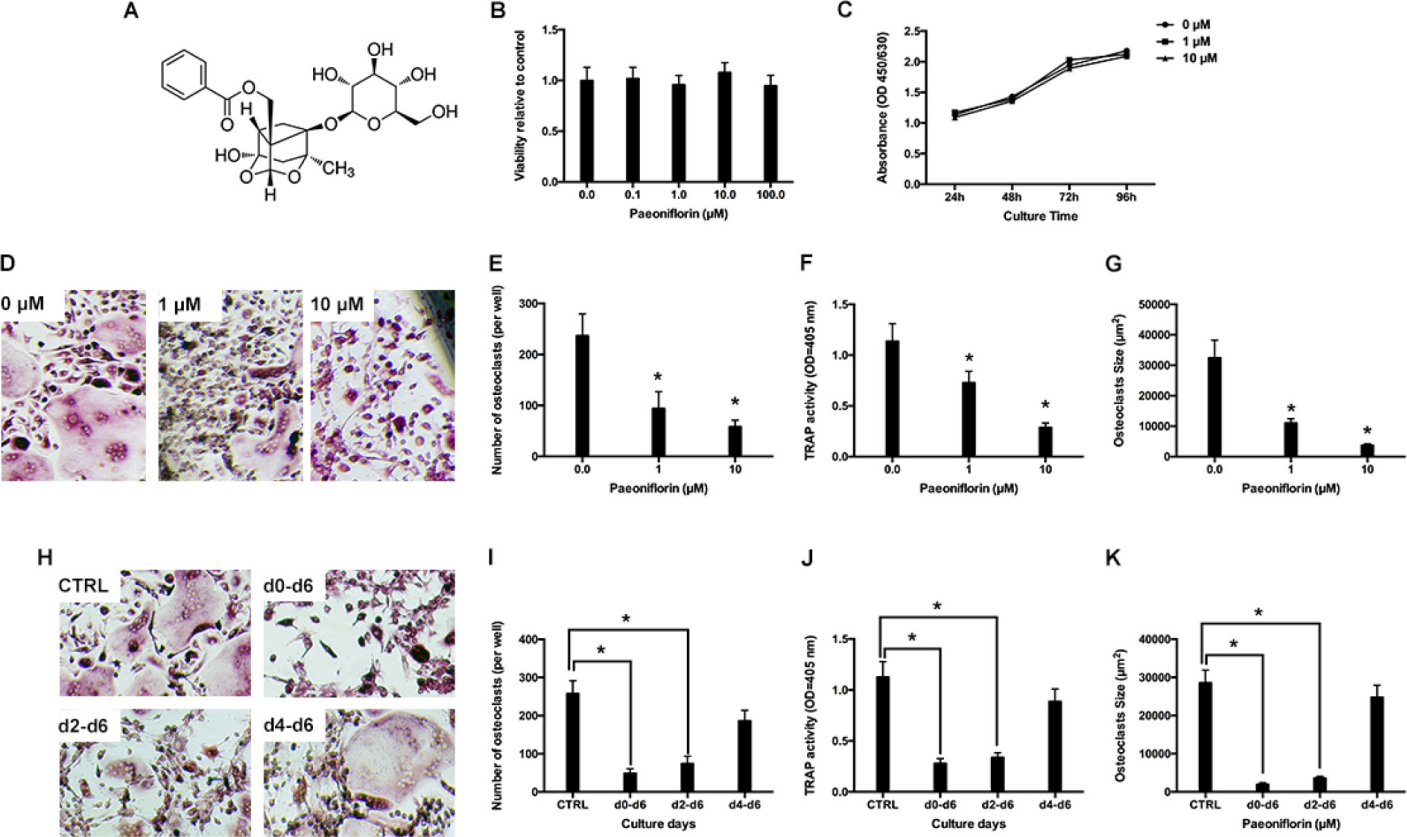
(**A**) Structure of paeoniflorin. (**B**) Cell viability, relative to the control, on treating BMMs with varying concentrations of paeoniflorin for 48 hours, as measured by the CCK8 assay. (**C**) Cell viability, relative to the control, on treating BMMs for prolonged duration with paeoniflorin (1 and 10 µM), as measured by the CCK8 assay (**D**) Representative light microscope images of RANKL-induced osteoclast formation when treated with indicated concentrations of paeoniflorin. (**E**) Count of multinucleated osteoclasts. (**F**) Analysis of osteoclast TRAP activity. (**G**) Analysis of osteoclast size. (**H**) Representative light microscope images of RANKL-induced osteoclast formation when treated with 10 μM of paeoniflorin on the indicated days. (**I**) Count of multinucleated osteoclasts. (**J**) Analysis of osteoclast TRAP activity. (**K**) Analysis of osteoclast size. Data are presented as mean ± SD; ^*^P < 0.05.

Various concentrations of paeoniflorin were used to treat bone-marrow derived macrophages (BMMs) for about 48 hours, and the resultant cell viability was estimated by using a CCK8 assay. Compared to control cells (that did not receive any treatment), paeoniflorin (up to 100 μM) did not result in any cytotoxic effects on cells (Figure 1B). Further, paeoniflorin at doses of 1 μM and 10 μM was applied for several days to analyze its cytotoxic effect. There was no paeoniflorin-induced inhibitory effect on BMM proliferation in the entire period of culture (Figure 1C).

### Paeoniflorin suppresses RANKL-evoked osteoclast differentiation

In the RANKL-induced osteoclastogenesis assay, BMMs were cultured together with M-CSF and RANKL, which facilitated the formation of numerous TRAP-positive multinucleated osteoclasts. Conversely, in the paeoniflorin-treated groups, osteoclast number, size, and TRAP activity were significantly suppressed (Figure 1D–1G). In the next step, we attempted to analyze the stage at which paeoniflorin inhibited osteoclast formation. Paeoniflorin was added to RANKL-induced osteoclastogenesis on specific days, and on the 6^th^ day, TRAP staining was performed. Paeoniflorin significantly inhibited osteoclast formation on day 0–6, suggesting that paeoniflorin mainly affected early osteoclastogenesis (Figure 1H–1J)

### Paeoniflorin suppresses osteoclast F-actin ring formation and bone resorption

**Figure 2:**
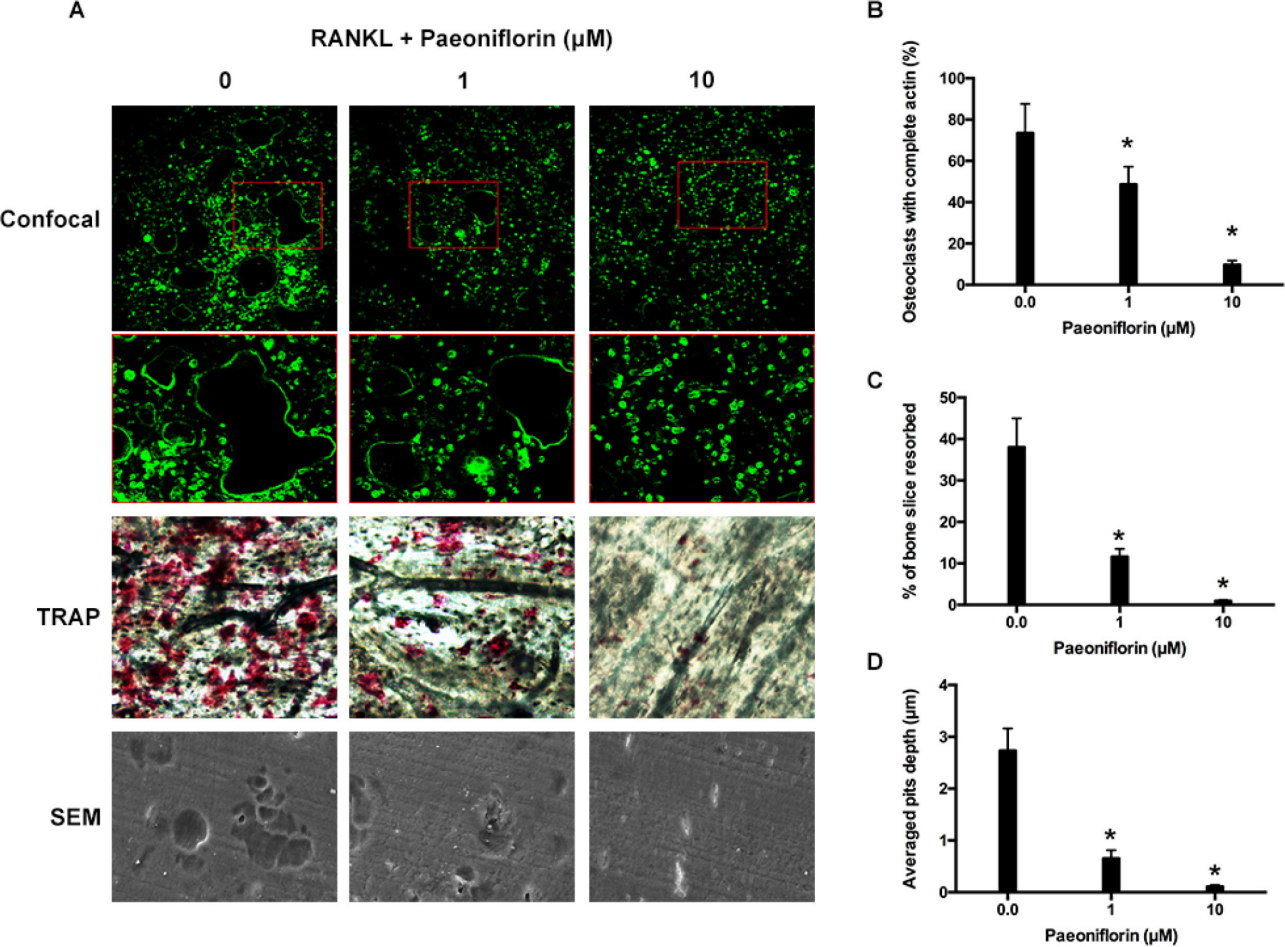
Paeoniflorin suppresses RANKL-induced osteoclast F-actin ring formation and bone resorption activity (**A**) Representative confocal images of osteoclasts stained for F-actin, TRAP stained bone slice, and SEM scanned bone slice in untreated osteoclasts, and osteoclasts treated with 1 μM and 10 μM of paeoniflorin. (**B**) Quantitative analysis of osteoclasts with complete actin. (**C**) Quantitative analysis of bone resorbed area. (**D**) Quantitative analysis of average pit depth. Data are presented as mean ± SD; ^*^P < 0.05.

Fibrous actin (F-actin) rings are typical cytoskeletal structures of osteoclasts that participate in bone resorption. The rings are characterized by dynamics and features [28]. The effects of paeoniflorin on F-actin ring formation was studies since the ring formation is aided by RANKL. The staining results, obtained by FITC (Fluorescein isothiocyanate)-labeled phalloidin, confirmed that RANKL (100 ng/mL) stimulation facilitated the formation of F-actin sealing ring, while paeoniflorin treatment in RANKL-stimulated (100 ng/mL) cells resulted in its suppression (Figure 2A and 2B). According to our results, paeoniflorin suppresses the formation of F-actin ring through suppressing osteoclastogenesis. Subsequently, resorption pit assay on dentin slice was used to examine whether the osteoclastic activity was damaged or not. RANKL-treated cells were found to have several resorption pits (Figure 2). Paeoniflorin appeared to significantly suppress the formation of resorption pits in RANKL-treated cells. To summarize, paeoniflorin suppressed the formation of osteoclast F-actin ring, and resulted in lesser bone resorption activity.

### Paeoniflorin inhibits the expression of RANKL-stimulated osteoclast-specific gene

**Figure 3:**
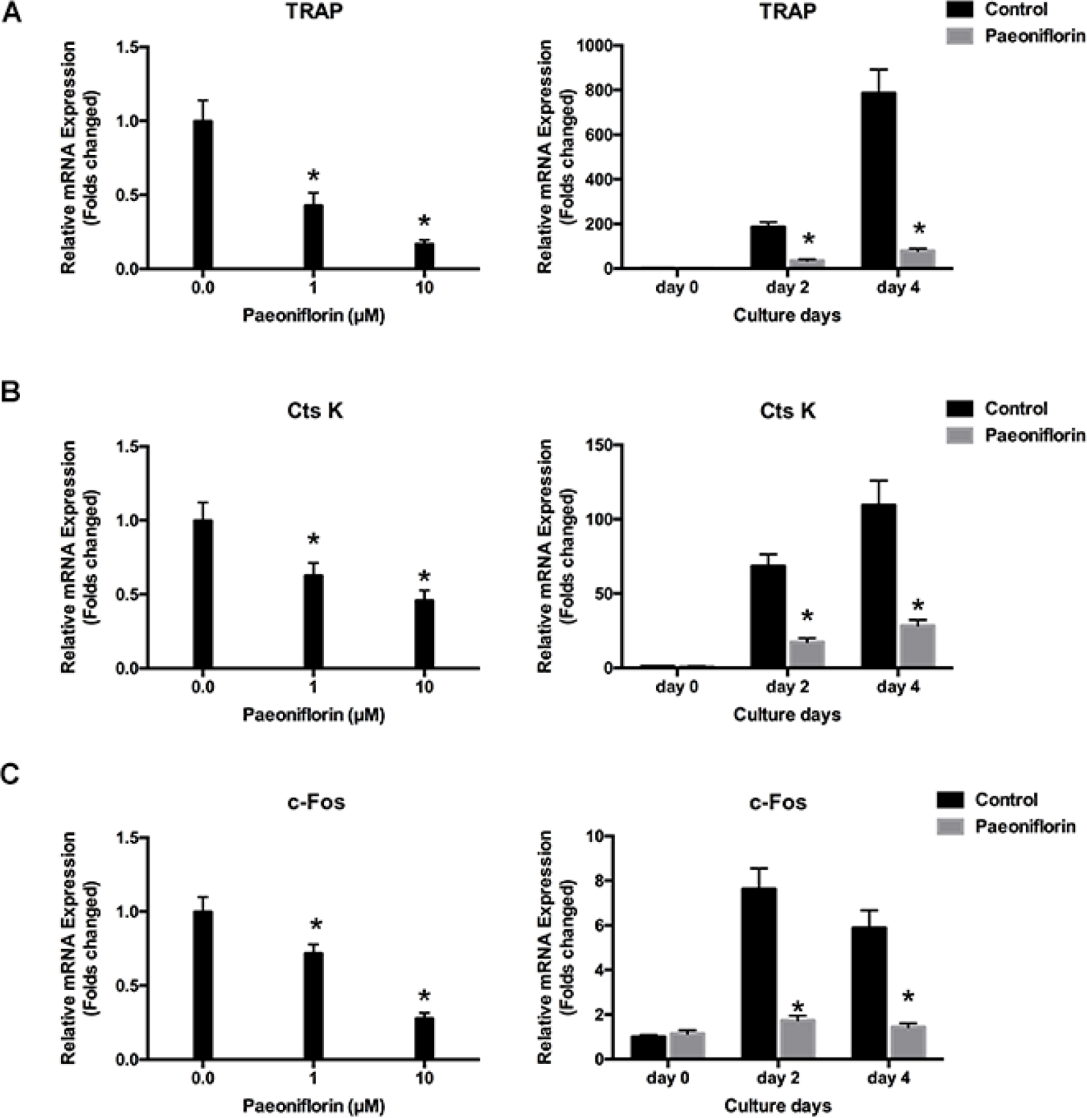
Paeoniflorin inhibits RANKL-evoked osteoclast gene expression (**A**) TRAP expression level, normalized by comparing with β-actin expression. (**B**) CtsK expression level, normalized by comparing with β-actin expression. (**C**) c-Fos expression level, normalized relative to β-actin expression. Data are presented as mean ± SD; ^*^P < 0.05.

Osteoclast differentiation and bone resorption result from the upward regulation of osteoclast-specific genes including TRAP, cathepsin K and c-Fos, which in turn are induced by RANKL. We investigated to determine whether paeoniflorin had any inhibitory effect on the expression of osteoclast-specific genes. Our results demonstrated that paeoniflorin treatment resulted in the inhibition of the RANKL-evoked mRNA expression of TRAP, cathepsin K, and c-Fos in a dose-dependent manner (Figure 3). These findings confirm the inhibitory effect of paeoniflorin on osteoclastogenesis and bone resorption.

### Paeoniflorin inhibits RANKL-evoked NF-κB activation

**Figure 4:**
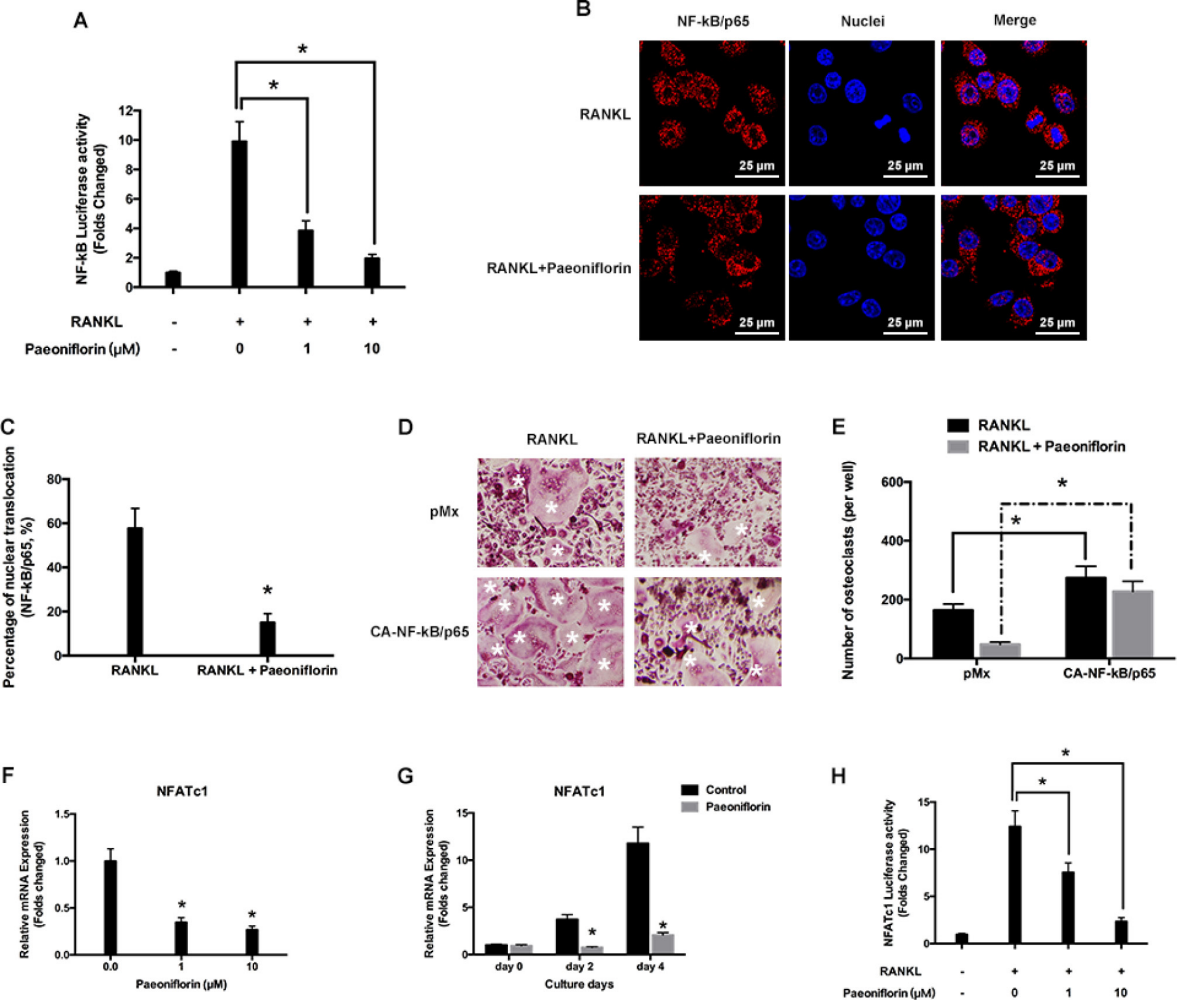
Paeoniflorin suppresses RANKL-evoked NF-κB activity and p65 nuclear translocation (**A**) Paeoniflorin inhibits RANKL-evoked NF-κB-dependent transcription activity. (**B**) Immunofluorescence analysis of the p65 nuclear trans localization. (**C**) Quantitative analysis of p65 nuclear trans localization. (**D**) BMMs infected with retroviruses harboring control pMX with Ca-NF-κB/p65 expression construct by using the retroviral method. Mature TRAP-positive multinucleated osteoclasts are seen on TRAP staining. (**E**) TRAP positive cells defined as osteoclasts (nuclei ≥ 3). (**F**) and (**G**) RANKL-evoked NFATc1 expression detected by real-time PCR assay. (**H**) Paeoniflorin suppresses RANKL-induced NFATc1-dependent transcription activity. Data are presented as mean ± SD; ^*^P < 0.05.

Paeoniflorin was found to inhibit NF-κB activation in a dose-dependent manner (Figure 4A). This was recognized based on the effect of paeoniflorin on RANKL-regulated NF-κB subunit p65 nuclear translocation. Pre-treatment with paeoniflorin appeared to slow down the nuclear translocation of p65 on RANKL stimulation, when compared to vehicle pre-treated control cells (Figure 4B, 4C). Further, BMMs were infected with NF-κB/p65-expressing retrovirus to analyze whether ectopic expression of NF-κB/p65 had the ability to reverse the inhibitory effect exerted by paeoniflorin on osteoclast differentiation. According to Figure 4D and Figure 4E, the ectopic expression of NF-κB/p65 resulted in osteoclast differentiation regardless of the presence of paeoniflorin. In addition, osteoclast specific transcript factor NFATc1 was found to be significantly downregulated by paeoniflorin, which confirmed the inhibitory effect of paeoniflorin on osteoclast formation and function at the molecular level (Figure 4F–4H). These findings suggest that NF-κB activity is the key pathway by which paeoniflorin exerts its effect on osteoclast differentiation. However, we cannot exclude the possibility that paeoniflorin may also have a controlling effect on other proteins in inhibiting osteoclast differentiation.

### Paeoniflorin has no inhibitory effect on osteoblast proliferation

**Figure 5:**
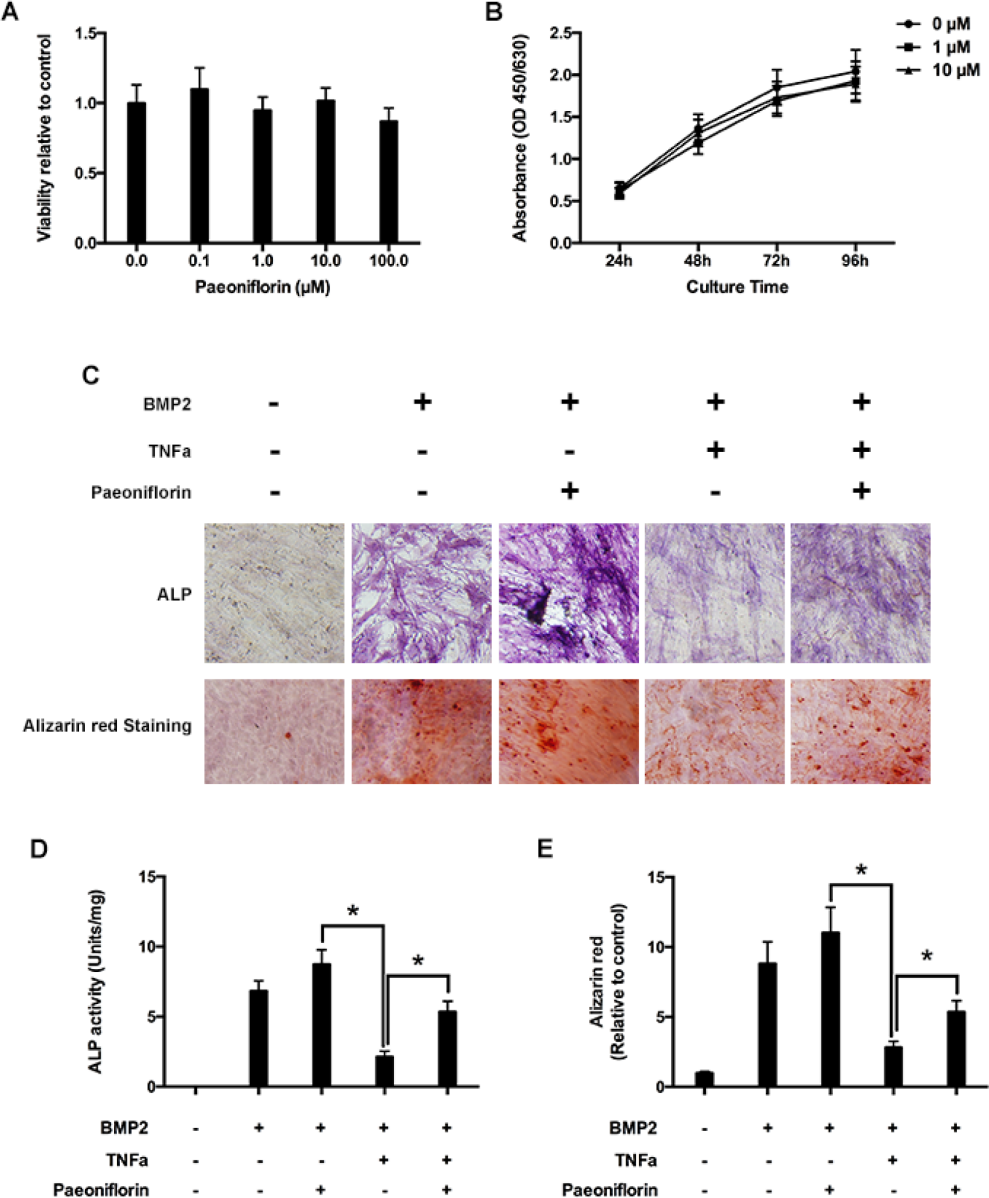
Paeoniflorin inhibits TNF-α-mediated suppression of osteoblast differentiation and mineralization (**A**) Cell growth estimation on treating osteoblasts with varying paeoniflorin concentrations for 48 hours, by using the CCK8 assay. (**B**) Cell growth estimation on treating osteoblasts with 1 μM and 10 μM of paeoniflorin on specific days, by using the CCK8 assay. (**C**) Cells, as seen on the day 14 of culture with ALP staining, and on the day 21 with Alizarin red S staining. (**D**) Numerical ALP activity in cell lysates on day 14. (**E**) Alizarin red quantitative analysis on day 21 of culture. Data are presented as mean ± SD; ^*^P < 0.05.

Primary osteoblasts were cultured in 96-well plates and cultured at different paeoniflorin concentrations for 48 hours, followed by the CCK-8 assay. Paeoniflorin was found to have no cytotoxic effects on osteoblast proliferation (Figure 5A). Furthermore, paeoniflorin had no inhibitory effects on osteoblast proliferation even on extended culture times at concentrations of 1 μM and 10 μM (Figure 5B).

### Paeoniflorin stimulates osteoblast differentiation and mineralization, and blocks TNF-α mediated suppression in osteoblastogenesis

We evaluated the effect of paeoniflorin on osteoblastogenesis, including early differentiation and late stage mineralization. We found that paeoniflorin significantly stimulated osteoblast differentiation, as identified by ALP staining (Figure 5C) and ALP activity analysis outcomes (Figure 5D). Furthermore, to study the impact of paeoniflorin on osteoblast mineralization, paeoniflorin-treated osteoblasts were stained with Alizarin Red S. Matrix mineralization, an indicator of late osteoblast differentiation, was found to be significantly enhanced by paeoniflorin (Figure 5C and 5E). Meanwhile, previous studies demonstrated that TNF-α-induced NF-κB activation has an anti-anabolic on osteoblast differentiation, and therefore, on bone creation [14, 29]. Thus, we investigated the effect of paeoniflorin on osteoblastogenesis in the presence of TNF-α. According to the ALP staining outcomes presented in Figure 5C, BMP-2 significantly stimulated osteoblast differentiation. This trend was not observed in the presence of TNF-α. The addition of paeoniflorin appeared to have a rescue effect on TNF-α-induced reduction of osteoblast differentiation. This trend was identified on ALP activity analysis (Figure 5D), indicating that paeoniflorin blocks TNF-α-mediated suppression of osteoblastogenesis. A similar trend was also identified on Alizarin Red S assay, indicating that paeoniflorin also blocks TNF-α-mediated suppression of late-stage osteoblast differentiation (Figure 5E).

### Paeoniflorin stimulates osteoblast-specific gene expressions

**Figure 6:**
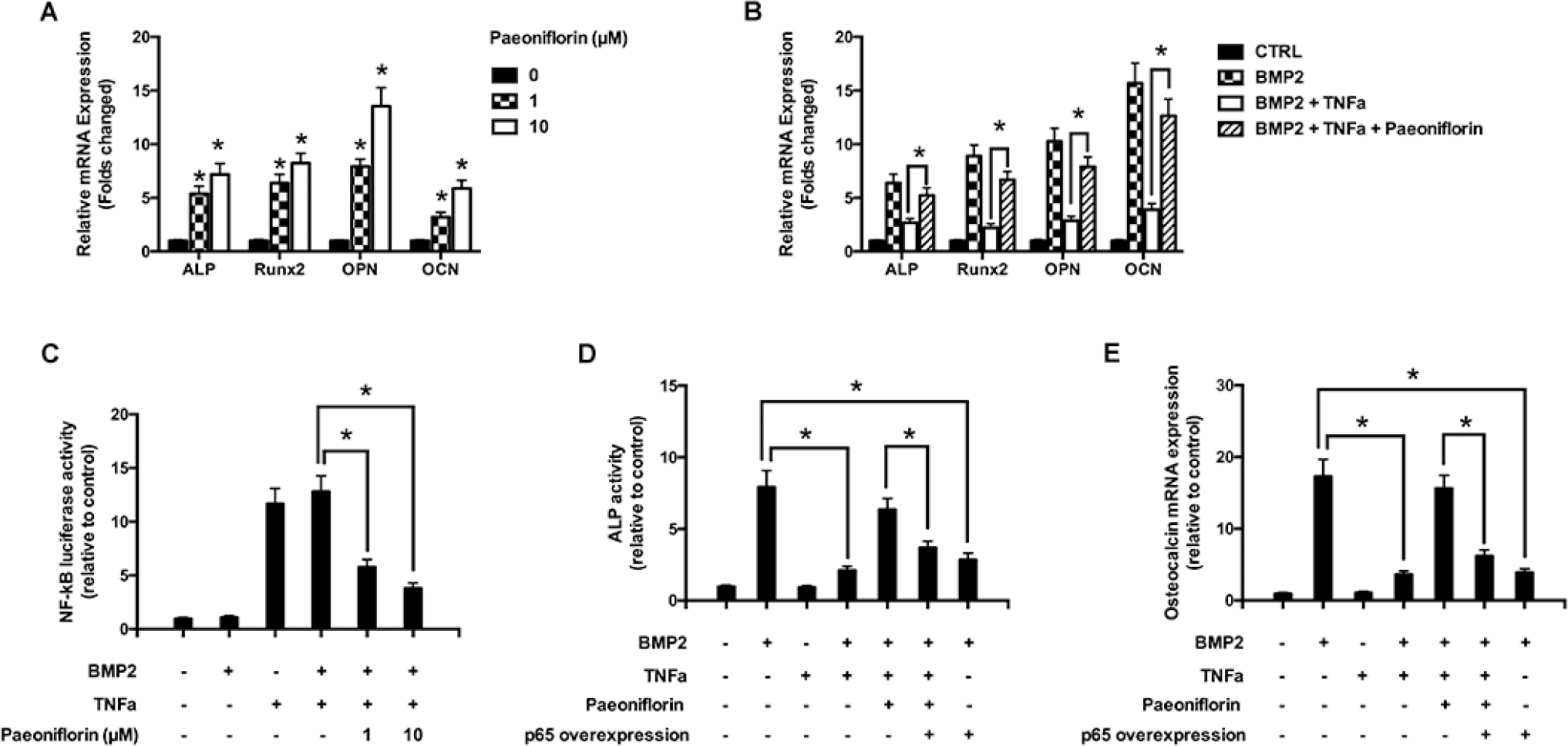
Molecular basis of the action of paeoniflorin (**A**) mRNA expression of OCN, OPN, Runx2, and ALP on day 14. (**B**) mRNA expression of OCN, OPN, Runx2, and ALP, with or without TNF-α, on day 14 (Quantitative real-time PCR, with the control group expression normalized to 1). (**C**) NF-κB luciferase activity on treatment with specific paeoniflorin concentrations. (**D**) ALP activity assay upon treatment with the indicated paeoniflorin concentration in the presence of p65 over-expression. (**E**) Osteoblast specific gene expression of OCN on treatment with the indicated paeoniflorin concentrations in the presence of p65 over-expression. Data are presented as mean ± SD; ^*^P < 0.05.

We further studied the effect of paeoniflorin on gene expression during osteoblastogenesis (Figure 6A). The mRNA of the primary osteoblast transcription factor Runx2 was found to be subjected to an upward regulation in a paeoniflorin dose-dependent method. This is consistent with enhanced osteoblast differentiation without affecting osteoblast characteristics. In addition, paeoniflorin appeared to improve osteogenic-specific gene expressions, including that of ALP, OCN, and OPN. We used real-time PCR (polymerase chain reaction) to study the influence of paeoniflorin on osteoblastogenesis in the presence of TNF-α at the gene expression level. As shown in Figure 6B, BMP-2 significantly upregulated the expression of osteoblast-specific genes compared to the negative control. This trend was not observed in the presence of TNF-α. However, the TNF-α-dependent reduction in osteoblast gene expression was efficiently rescued by the addition of paeoniflorin. These results indicate the ability of paeoniflorin in blocking TNF-α-mediated suppression of osteoblastogenesis at the gene level.

### Paeoniflorin downregulates TNF-α-mediated stimulation of NF-κB activity

The luciferase assay was used to evaluate the role of paeoniflorin in inhibiting TNF-α-induced NF-κB activity (Figure 6C). Our results clearly demonstrated that TNF-α treatment resulted in an increase in NF-κB luciferase activity in osteoblasts. Paeoniflorin treatment significantly weakened the luciferase activity stimulation, indicating that it influenced the suppression of NF-κB activation in a dose-dependent manner. Our findings indicate that paeoniflorin suppresses NF-κB signaling in osteoblasts. The ALP assay identified that p65 over-expression induced the down-regulation of paeoniflorin-mediated ALP activity (Figure 6D). Furthermore, to verify if the rescue of NF-κB activity affected osteoblast differentiation, we studied the expression of osteoblast-specific genes. Consistent with our previous results, paeoniflorin appeared to significantly preserve OCN gene expression. However, in the set of cells that overexpressed p65, paeoniflorin was unable to rescue OCN expression efficiently (Figure 6E). These findings indicate that the mechanism of action of paeoniflorin on osteoblast differentiation involves inhibition of NF-κB stimulation.

### Paeoniflorin increased BMD of OVX rats (rats that underwent ovariectomy)

**Figure 7:**
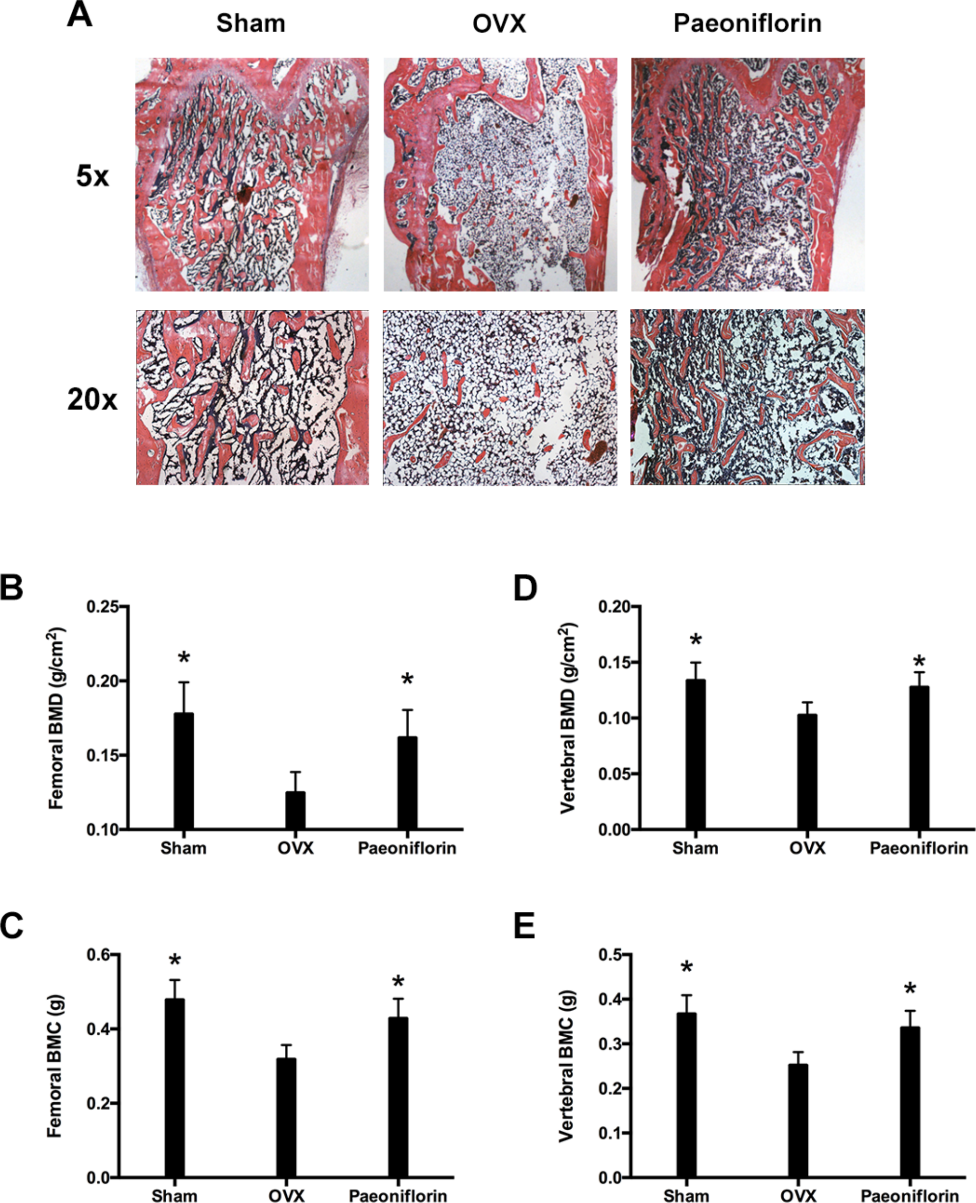
Treatment effects of paeoniflorin on OVX rats (**A**) HE staining of bone tissues. (**B**) Bone mineral density (BMD) of right femur. (**C**) Bone mineral content (BMC) of right femur. (**D**) Bone mineral density (BMD) of lumbar vertebrae. (**E**) Bone mineral content (BMC) of lumbar vertebrae. Data are presented as mean ± SD; ^*^P < 0.05.

Rats in the sham group were found to have uniform, plump, continuous, dense, regular, rich, brown, and well-formed trabecular bone (Figure 7A). The bone marrow cavity was small with abundant marrow cells in the cavity, and few fat droplets. In comparison, the rats in the OVX (ovariectomy) group were found to have trabecular bone that was significantly reduced, sparse, irregular, and partially fractured. The connectivity between the trabecular bones was poor and contained a large number of ceca, and the bone marrow cavity was enlarged with few hematopoietic cells and several fat droplets. However, in the paeoniflorin treated group, the protective effects of paeoniflorin on OVX-induced bone loss were confirmed by histological examination. Micro-CT (computed tomography) analyses demonstrated the transformation in the BMC (bone mineral content) and BMD (bone mineral density) of the right femur and lumbar vertebrae (L3–L4) (Figure 7). The BMC and BMD of the right femur and lumbar vertebrae were significantly weakened by OVX. Treatment with paeoniflorin increased the BMC and BMD of the right femur and lumbar vertebrae in the OVX rats.

### Improvement of bone trabecular microarchitecture of OVX rats by Paeoniflorin

**Figure 8:**
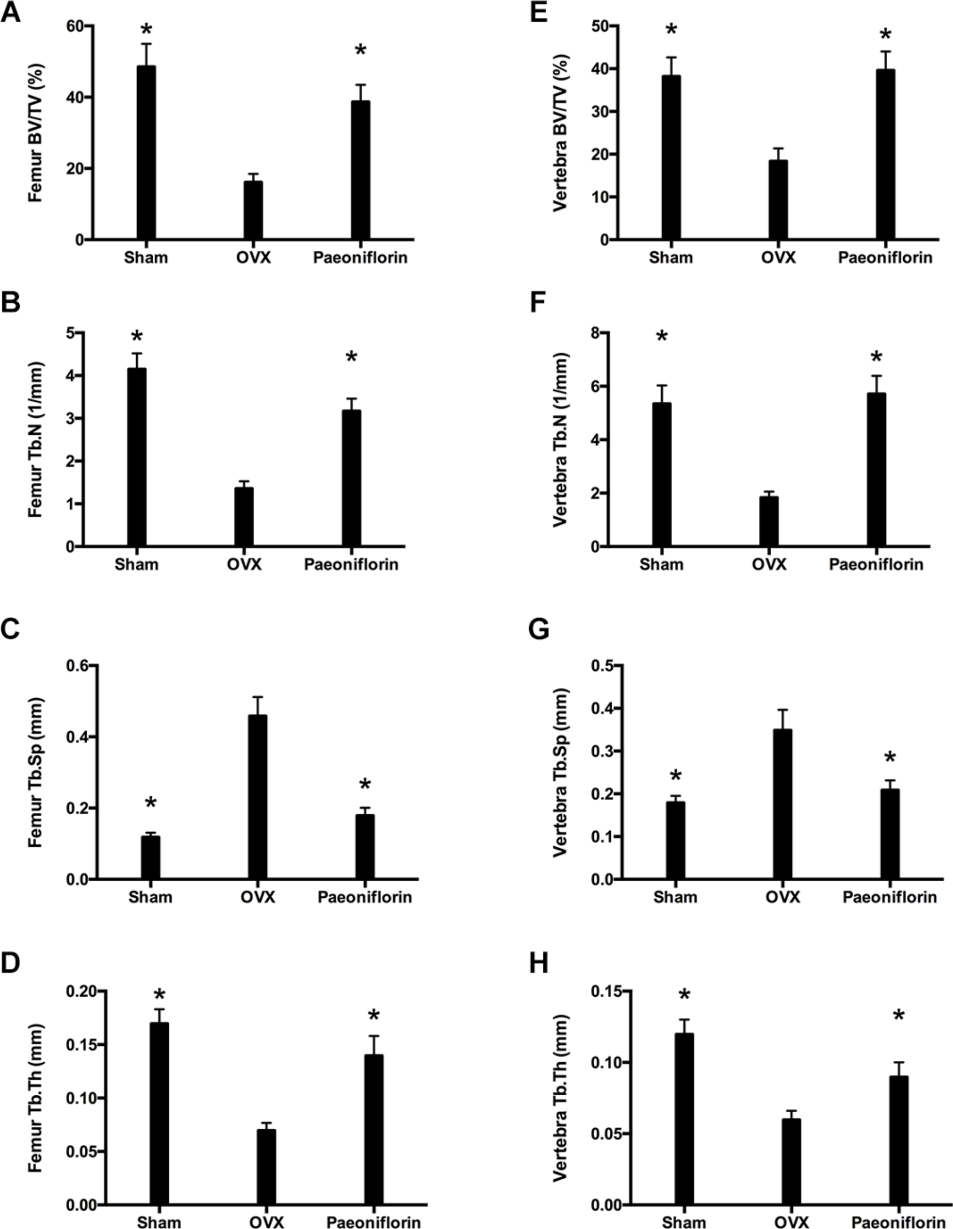
Effect of paeoniflorin on trabecular microarchitecture: Micro-CT images (**A**–**D**) Bone volume per tissue volume (BV/TV) ratio, trabecular thickness (Tb.th), trabecular number (Tb.N.), and trabecular separation (Tb.Sp.) of right femur. (**E**–**H**) Bone volume per tissue volume (BV/TV) ratio, trabecular thickness (Tb.th), trabecular number (Tb.N.), and trabecular separation (Tb.Sp.) of lumbar vertebrae. Data are presented as mean ± SD; ^*^P < 0.05.

Treatment with paeoniflorin resulted in a significant improvement in the bone trabecular microarchitecture (Figure 8). Micro-CT demonstrated that the trabecular parameters of micro-architecture (bone volume per tissue volume [BV/TV], trabecular thickness [Tb.th], and trabecular number [Tb.N.]) were poorer in OVX rats when compared to the sham control. However, these values for femur and vertebra improved significantly with paeoniflorin treatment. An improvement was also noted in the parameter, Tb.Sp (trabecular separation), with paeoniflorin treatment.

### Paeoniflorin improved the bone strength of OVX rats

**Figure 9:**
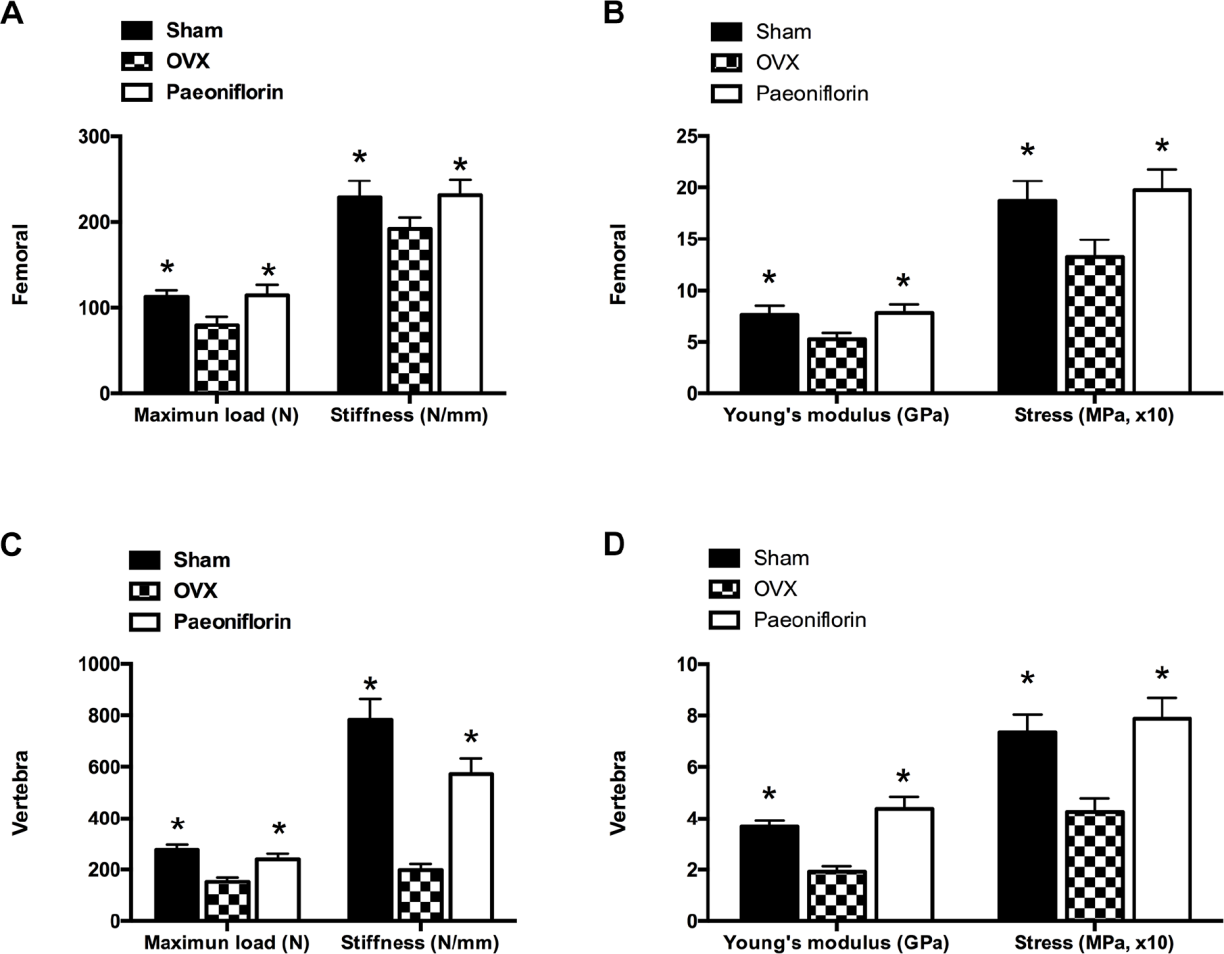
Effect of paeoniflorin treatment on the biomechanical properties of bone (**A**, **B**) Femoral and (**C**, **D**) vertebral maximum load (N), maximum stress (MPa), and Young’s modulus (GPa) and stiffness (N/mm; the slope of the linear region). Data are presented as mean ± SD; ^*^P < 0.05.

The *ex vivo* three-point bending test and axial compression test were used to examine the biomechanical competence of the femoral diaphysis and lumbar vertebral bodies. There was a dramatic decrease in the Young’s modulus and ultimate stress in the femur and lumbar vertebrae following OVX, when compared to the sham group. Treatment with paeoniflorin improved the Young’s modulus significantly, in addition to reversing the reduction in the ultimate stress of the bones caused by OVX. Furthermore, paeoniflorin also appeared to rescue the decrease in maximum load and stiffness resulting from OVX (Figure 9).

### Paeoniflorin ameliorated biochemical parameters of OVX rats

**Figure 10:**
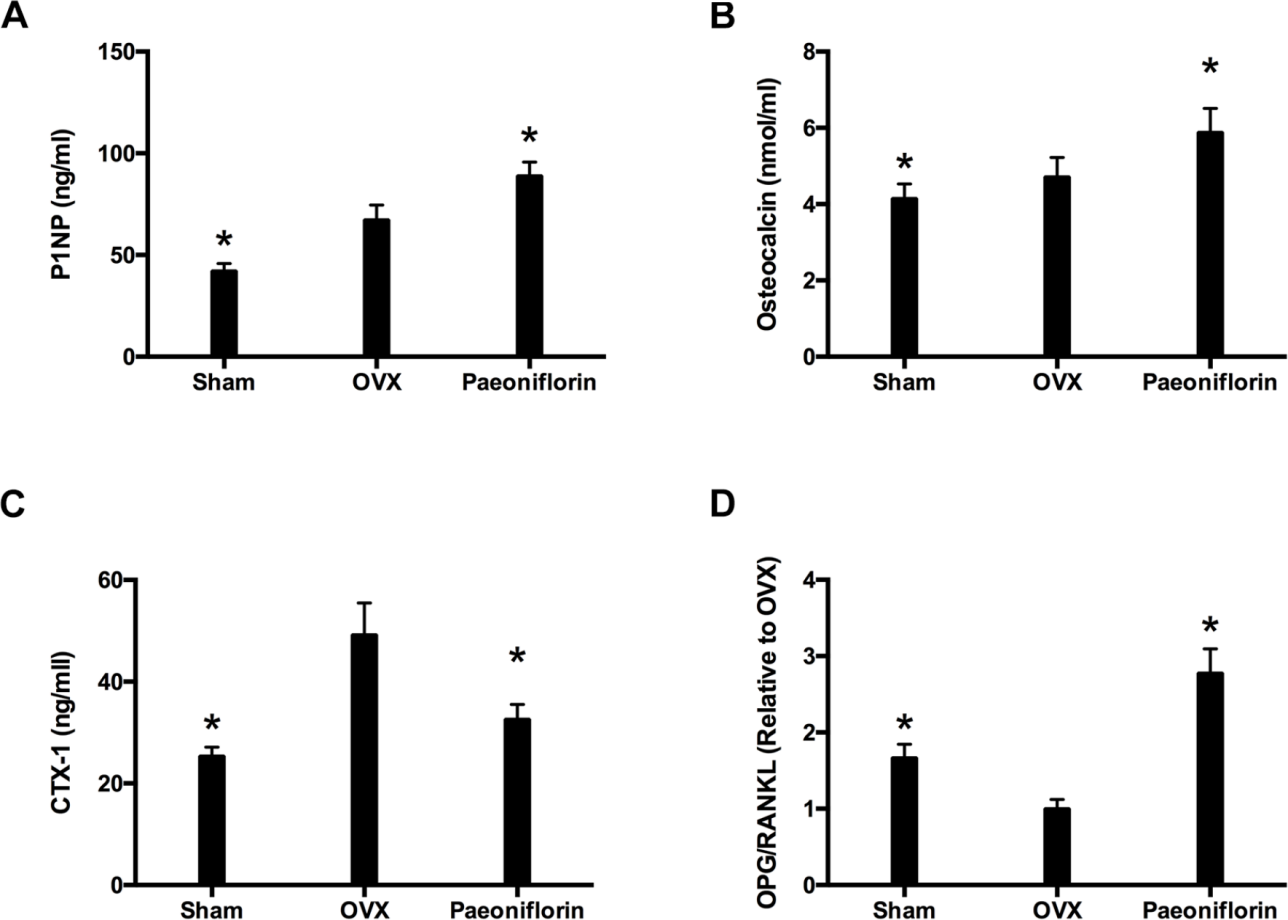
Effect of paeoniflorin treatment on bone turnover biomarkers in OVX rats (**A**) P1NP. (**B**) Osteocalcin. (**C**) Serum CTX-I. (**D**) mRNA expression ratio of OPG/RANKL. Data are presented as mean ± SD; ^*^P < 0.05.

The markers for bone resorption and formation were analyzed following the completion of paeoniflorin treatment. Paeoniflorin treatment appeared to inhibit the serum CTX-I level (Figure 10C). Similarly, there was a significant increase in the levels of OC and P1NP with paeoniflorin (Figure 10A and 10B), when compared to the OVX group. Figure 10D demonstrates the expression of OPG and RANKL mRNA in OVX rats that were treated with paeoniflorin. The OPG/RANKL mRNA expression ratio was significantly reduced in OVX rats when compared to the sham group. However, treatment with paeoniflorin resulted in a significant increase in the OPG/RANKL ratio in OVX rats. Our results demonstrating the effect of paeoniflorin on OPG and RANKL expressions as well as on the OPG/RANKL ratio, indicate that paeoniflorin suppresses osteoclastogenesis.

## DISCUSSION

Bone remodeling involves the two phases of bone formation and bone resorption. These two phases are linked by coupling factors including RANKL that maintain bone homeostasis. Since these processes are connected, drugs like bisphosphonates that weaken bone degradation, also result in decreased bone formation. Similarly, drugs like parathyroid hormone that improve bone synthesis also result in increased bone degradation. Consequently, there is a search for newer dual-action therapeutic agents for osteoporosis that simultaneously suppress bone resorption and accelerate bone formation.

It is known that TNF-α blocks osteoblast differentiation and stimulates osteoclast formation, and results in impaired new bone deposition, and accelerated bone resorption, especially in conditions like inflammatory bone diseases and osteoporosis [30]. Furthermore, TNF-α has also been demonstrated to be a pivotal factor in inflammation-linked bone degeneration, wherein it is known to damage normal bone recreation (biphasic effects of enhanced bone reabsorption and impaired bone creation) [31]. The mechanism by which TNF-α suppresses osteoblast differentiation and causes impaired bone creation may involve the NF-κB signaling pathway [14, 29]. Therefore, the recovery of the balance between bone reabsorption and bone creation is crucial for effectively managing inflammatory bone diseases and osteoporosis. As biphasic factors, TNF-α and NF-κB are potential targets for novel drug design, and their suppressors can potentially be used to treat inflammatory bone diseases and osteoporosis [9].

In this study, we analyzed the role of paeoniflorin in altering BMP-2-induced osteoblast differentiation, and in attenuating TNF-α-induced suppression of osteoblast differentiation. Mouse primary osteoblasts were stimulated with BMP-2 to undergo differentiation. ALP activation assay and Alizarin Red S staining revealed a significant stimulatory effect of paeoniflorin on osteoblastogenesis. Furthermore, paeoniflorin was found to have a significant rescue effect on TNF-α-induced suppression of osteoblast formation and mineralization. Moreover, analysis of gene expression of the transcription factor Runx2 demonstrated that osteoblast marker gene expression was protected from TNF-α-induced suppression by paeoniflorin. These results indicate that paeoniflorin restores osteoblast differentiation by preventing the inhibitory action of TNF-α. Paeoniflorin was also found to enhance the expression of osteogenic-specific genes ALP, OCN, and OPN in a dose-dependent manner. We further analyzed the role of paeoniflorin on gene expression. BMP-2 significantly upregulated osteoblast-specific gene expression when compared to the negative control, but this effect was lost in the presence of TNF-α. The addition of paeoniflorin reversed this TNF-α-induced suppression of osteoblast gene expression effectively, indicating the ability of paeoniflorin to block the suppression of osteoblastogenesis at the gene level. We investigated further to determine if the suppression of NF-κB activation could explain the effect of paeoniflorin on osteoblast differentiation. Results from the luciferase assay suggested that NF-κB activation was suppressed by paeoniflorin treatment. Meanwhile, the over-expression of NF-κB subunit p65 prevented paeoniflorin-induced osteoblastogenesis, confirming that paeoniflorin modulated osteoblast differentiation by suppressing NF-κB. Osteoblast gene expression analysis further supported this observation. Our results demonstrate that paeoniflorin influences osteoblast differentiation by inhibiting the NF-κB pathway.

During the process of osteoclastogenesis, the recruitment of the adaptor molecules, the TNF receptor-associated factors (TRAFs), is dependent on the connection between RANKL and its receptor, RANK. NF-κB signaling pathway is one of the key downstream signaling pathways in this complex [32]. Paeoniflorin was found to have a significant influence on the transcriptional activity of NF-κB, which was confirmed by the downregulation of NF-κB subunit p65 nuclear translocation. c-Fos is also activated by RANKL and has a synergistic relationship with NF-κB in evoking NFATc1 and activating osteoclast-specific gene transcription. Our results demonstrate that paeoniflorin influences the signaling pathway of NF-κB in several ways, leading to the suppression of NFATc1 activation, and therefore, of osteoclastogenesis.

Further, we also demonstrated that paeoniflorin inhibited the transcriptional activity of NF-κB and the translocation of its subunit p65, confirming the role of paeoniflorin in regulating the NF-κB/NFATc1 signaling pathway in RANKL-evoked osteoclastogenesis. Since the gene expression for osteoclast differentiation and function is regulated by NFATc1, it can be concluded that paeoniflorin suppresses osteoclastogenesis-related marker gene expression including that of c-Fos, cathepsin K, and TRAP.

Our study demonstrated the protective effect of paeoniflorin on bone metabolism with the help of an ovariectomized (OVX) mouse model. Osteoporosis involves bone mass loss, which results in fragility fractures. BMD is an indicator of osteoporosis and fracture risk [33]. A few studies have demonstrated the role of paeoniflorin in improving BMD following ovariectomy. Based on the micro-CT findings of the trabecular bone structure, our study demonstrated that paeoniflorin may prevent trabecular bone structure deterioration following OVX. Further, its role in modifying bone structure, including the addition and redistribution of bone mass, may have an impact on the biomechanical properties of bone. The state of the bone is described by parameters that include extrinsic (ultimate force and stiffness) and intrinsic biomechanical properties (Young’s modulus and ultimate stress) [34, 35]. According to our data, paeoniflorin prevented loss of bone strength following OVX, as identified by the improvement in all bone biomechanical properties including ultimate force, stiffness, Young’s modulus, and ultimate stress. This implies that paeoniflorin may satisfy the requirements of an ideal therapeutic agent for osteoporosis [34]. Our findings also identified that the improvement in biomechanical properties resulted from the optimization of the trabecular bone structure.

Apart from analyzing the influence of paeoniflorin on the density, structure, and strength of bone, we also studied its effects on biochemical markers and bone histology. These findings may explain the potential mechanisms of the anti-osteoporotic effect of paeoniflorin. Serum OC and P1NP levels were considered as the principal biomarkers of the bone formation rate, while serum CTX-I was considered as the main biomarker for estimating the bone resorption rate. Our results indicated that Paeoniflorin resulted in lowered serum CTX-I, and increased serum OC and P1NP. These findings confirmed that paeoniflorin influenced bone metabolism by improving bone formation, and inhibiting bone resorption. In order to maintain skeletal integrity, the coordination between the osteoblasts and osteoclasts is very important. RANKL, activator of NF-κB receptor (RANK), and OPG regulate osteoclastogenesis with the help of immature cells of osteoblastic lineage. OPG is a decoy receptor with the potential to suppress RANKL activation, and therefore osteoclastogenesis. This has the effect of weakening the process of bone resorption [36]. Therefore, the ratio of OPG/RANKL expression is an important determinant of osteoclastogenic activity. Our study identified that paeoniflorin resulted in an increase in the OPG/RANKL expression ratio. These results indicate that paeoniflorin influences osteoclast differentiation by increasing the OPG/RANKL ratio.

**Figure 11:**
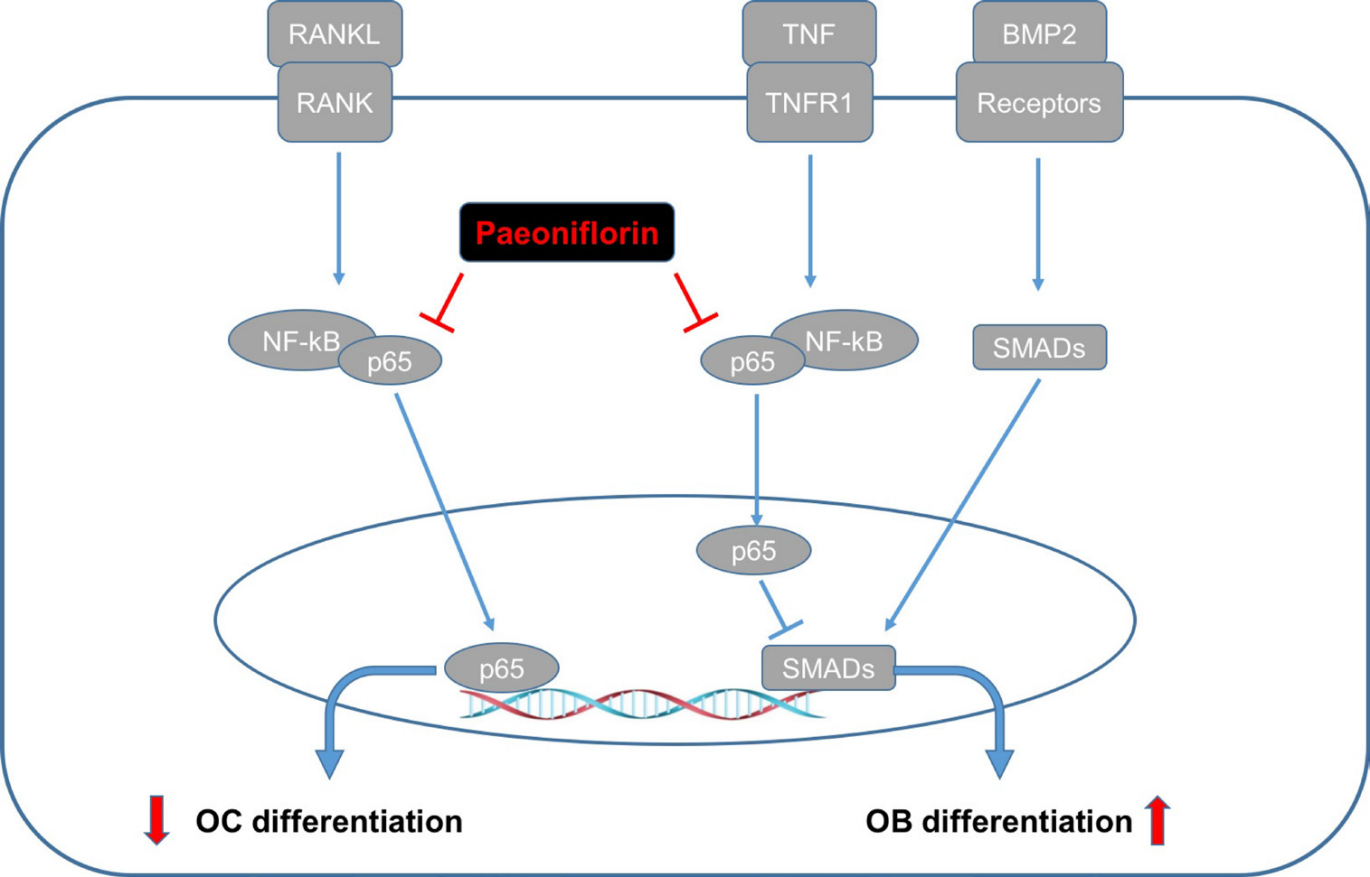
Graphical representation of the dual functional role of paeoniflorin on osteoblastogenesis and osteoclastogenesis

To summarize, our study is the first one to demonstrate that paeoniflorin influences bone remodeling by simultaneously suppressing bone resorption and improving bone formation. Paeoniflorin was found to inhibit osteoclast formation and bone resorption in a dose-dependent manner. It was also found to play a vital role in bone formation and mineralization. Further, paeoniflorin was also found to rescue TNF-α-mediated inhibition of osteoblastogenesis. The mechanism of action of paeoniflorin in maintaining bone homeostasis is markedly different from the other drugs used for osteoporosis such as bisphosphonates and PTH1-34, which either inhibit or stimulate the twin processes of bone resorption and formation [6, 36] (Figure 11). The bi-functional influence exerted by paeoniflorin makes it an attractive candidate for the design of novel alternative agents for the treatment and prevention of osteoporosis.

## MATERIALS AND METHODS

### Materials

The biological agents and reagents used in the study were the following: Paeoniflorin (C_23_H_28_O_11_; Molecular weight [MW], 480.45; purity, 98.78% [HPLC]) from Nanjing GOREN BIO Technology Co., Ltd., Nanjing, China; Dulbecco’s improved Eagle’s medium (DMEM), Fetal bovine serum (FBS), penicillin, and streptomycin from Hyclone, Thermo Fisher Scientific, Logan, UT, USA; recombinant mouse M-CSF, RANKL, BMP-2, and TNF-α from Peprotech, Inc., Rocky Hill, NJ, USA; cell counting kit-8 (CCK-8) from Dojindo Molecular Technologies Inc., Kumamoto, Japan; acid phosphatase, and leukocyte (TRAP) assay kit from Sigma (Sigma-Aldrich, US; anti-p65 antibody from Cell Signaling Technology, Beverly, MA, USA. All the reagents and agents were commercially procured. MH Zheng (Professor, University of Western Australia, Nedlands, WA 6009, Australia) provided cells that were transfected with luciferase reporter constructs and under regulation of NF-κB binding promoter elements. Figure 1 illustrates the molecular structure of Paeoniflorin.

### Cell culture

The mouse monocyte/macrophage precursors were separated from the bone marrow of 4-week-old C57BL/6 mice, and those precursors were divided into bone marrow derived monocytes (BMMs) in 30 ng/mL M-CSF. Cultivation of OC-like RAW264.7 cell line (ATCC, TIB-71) was conducted in complete a-MEM. A humid environment of 5% CO_2_ at 37°C was maintained to cultivate all cells. Mouse primary osteoblasts were grown and treated as previously described [37]. Briefly, primary osteoblasts were collected from the calvarias of postnatal day 1 mice that were processed by trypsin and collagenase. Osteoblasts were cultured in a-MEM that was replenished by FBS (10%), penicillin (50 U/mL), and streptomycin (50 mg/mL) in an environment of 5% CO_2_ at 37°C.

### Cytotoxicity analyses

Cytotoxicity analyses was conducted by using the Cell Counting Kit-8 according to manufacturer instructions. This was performed to estimate the influence of paeoniflorin on the ability of osteoblasts and osteoclasts to survive. Briefly, 96-well plates were used to cultivate cells irrespective of the paeoniflorin concentration. A GENios microplate reader (Tecan, Austria) was used to measure the optical density at 450 nm following 2 hours of culture in the CCK-8 solution. Cell viability was expressed as a percentage of the control.

### Osteoclastogenesis assay *in vitro*

96-well plates were used to culture BMMs overnight, each well containing 1 × 10^4^ cells along with a-MEM and M-CSF. The following day, 50 ng/mL of RANKL was used to stimulate BMMs, after ensuring adequate paeoniflorin concentration. The plates were fixed with 4% paraformaldehyde in phosphate-buffered saline (PBS) for about 10 min, and then stained for TRAP. TRAP-positive multinucleated cells that carried three or more nuclei were identified as osteoclasts. TRAP enzyme activity in cell lysates was quantified by surveying absorbance at 405 nm, and normalizing it against total protein according to the Bradford assay, by using an acid phosphatase kit (Sigma, USA).

### Actin ring formation and bone resorption assay

Actin ring formation assays and resorption assays were performed as previously described [38, 39]. Pre-osteoclasts were seeded on bone slices in 96-well plates at a density of 8 × 10^3^ cells/well with three replicates for 5–7 days. Three fields of view were randomly selected for each bone slice for further analysis. Wells were imaged by using a laser microscope (Olympus LEXT OLS4100, Melville, NY, USA) for the visualization of the erosion area. The resorption areas were analyzed on ImageJ (NIH, Bethesda, MD, USA). Three random fields were imaged for the measurement of the resorption areas.

### RNA extraction, reverse transcription and PCR

An RNeasy Mini Kit (QIAGEN, Hilden, Germany) was used to separate the total RNA from cultivated cells according to the manufacturer instructions. About 2 mg of the total RNA was translated into cDNA by employing M-MLV-RT and Oligo-dT primers (Promega) in 20 μL of RT buffer. Triplicate PCR reactions were carried out on 1 μL aliquots of cDNA. Each reaction was continued for 30 cycles: 94°C for 40 s, and proper annealing temperature (Ta) for 40 s and 72°C for 40 s. Electrophoresis was used for the analyses of PCR products in agarose gel. The expression of each gene was normalized to that of the b-actin housekeeping gene. As shown below, Ta and primer sets were always applied: murine b-actin: forward, 5′-AGCCATGTACGTAGCCATCC-3′ and reverse, 5′-CT CTCAGCAGTGGTGGTGAA-3′; and cathepsin K (CTSK): forward, 5′-GGGAGAAAAACCTGAAG-3′ and reverse, 5′-ATT CTGGGGACTCAGAGAGC-3′; and NFATc1: forward, 5′- CCGTTGCTTCCAGAAAATAA CA-3′ and reverse, 5′- TGTGGGATGTGAACTCGGAA-3′; and TRAP: forward, 5′-CTGGAGTGCACGATGCCA GCGACA-3′ and reverse, 5′-TCCGTGCTCGGCGATGG ACCAGA-3′; and c-Fos: forward, 5′- CCAGTCAAGA GCATCAGCAA-3′ and reverse, 5′- AAGTAGTGCAG CCCGGAGTA-3′; and ALP: forward, 5′-TGACCTTCT CTCCTCCATCC-3′ and reverse, 5′-CTTCCTGGGA GTCTCATCCT-3′; and OCN: forward, 5′-CTTGAAGAC CGCCTACAAAC-3′, 5′-GCTGCTGTGACATCCATA C-3′; and BSP: forward, 5′-AGGACTGCCGAAAGGA AGGTTA-3′ and reverse, 5′-AGTAGCGTGGCCGGTACT TAAA-3′; and Runx2: forward, 5′-TCCTGTAGATCC GAGCACCA-3′ and reverse, 5′-CTGCTGCTGTTGTT GCTGTT-3′; and OPN: forward, 5′-TGTTCCTACCAA GATTATACCAAAT-3′ and reverse, 5′-CGCTCGATTTG CAGGTCTTT-3′.

### Luciferase reporter gene activity assay

The influence of paeoniflorin on RANKL-induced NF-κB and NFATc1 activation was studied by using RAW264.7 cells, which were transfected with NF-κB luciferase reporter construct [40, 41]. Briefly, 48-well plates were used for cell culture, wherein cells were left in the cell cultivation media for 24 hours. Subsequently, the cells were subjected to preliminary treatment for 1 hour irrespective of the paeoniflorin concentrations. The cells were exposed to RANKL (50 ng/mL) for 8 hours. A Promega Luciferase Assay System (Promega, Madison, WI, USA) was used to analyze the luciferase activity, which in turn was normalized to the activity of the vehicle control.

### NF-κB localization by using confocal microscopy

Prior to treatment, cells were seeded on cover slips and were hungry for 24 hours. Subsequently, paeoniflorin was applied to treat cells for 4 hours, while RANKL (100 ng/mL) was used to stimulate cells for about 30 min. Then, cells were penetrated by 0.1% Triton-X 100 and stopped in 3% BSA for 1 hour under indoor temperature. The antibodies of the NF-κB subunit p65 (1:100) were cultured for about 12 hours at 4°C. For nuclear staining, DAPI solution (Sigma-Aldrich) was added to the culture for about 5–10 min in the absence of sunlight. The images of nuclear translocation of p65 were obtained by employing a confocal system (Nikon, Tokyo, Japan). The mean intensity of the fluorescence was measured using Leica Confocal Software, for a minimum of 200 cells per condition.

### Retrovirus preparation and infection

Retrovirus packaging and infection was performed by using X-tremeGENE 9 (Roche, Nutley, NJ, USA), with the retroviral vectors pMX-constitutively active (CA)-NF-κB/p65-IRES-EGFP, according to the manufacturer’s protocol. Briefly, packaging was performed by transient transfection of these pMX vectors into Plat-E retroviral packaging cells. The cultivated supernatants of the retrovirus-producing cells were assembled after supernatants were incubated in new medium for about 2 days. For retroviral infection, BMMs were cultivated in the presence of M-CSF (20 ng/mL) for two days, and were simultaneously incubated with viral supernatant pMX-CA-NF-κB/p65-IRES-EGFP virus-making Plat-E cells, accompanied by polybrene (10 mg/mL) and M-CSF (20 ng/mL) for 6 hours. The infection efficiency of the retroviruses was confirmed to be in excess of 80% based on the expression of the green fluorescent protein (GFP). In the presence of M-CSF (20 ng/mL) and RANKL (100 ng/mL), BMMs were allowed to differentiate for 4 days following their infection. TRAP staining was used to detect osteoclast formation. To prevent adenovirus infection, homologous recombination between cosmid cassette expression and parental virus genome in 293 cells was used to build recombinant adenoviruses of the wild-type (Ad-LacZ) by using an adenovirus construction kit (Takara, Kyoto, Japan). According to experiment results, due to the absence of E1A-E1B, the viruses only keep 393 cells rather than any proliferative activity. A regulated point assay was used to determine virus titers.

### ALP staining and activity assay

For the ALP staining assay, we used 1 × 10^5^ cells per well of a 24-well plate. Additionally, cells were cultured for 14 days for the ALP staining assay. Lysate alkaline phosphatase (ALP) activity was analyzed by applying p-nitrophenolphosphate as the substrate at a pH of 10.2, wherein the yellow product was measured as absorbance at 405 nm. In the process of cell cultivation, naphthol phosphate substrate and fast violet B were used to detect ALP by making the product more apparent at pH 9.5 (alkaline phosphatase kit, Sigma, St. Louis, MO).

### Staining and mineralization of alizarin red S

For the mineralization assay, we used 1 × 10^5^ cells per well of a 24-well plate. Additionally, cells were cultured for 21 days for the mineralization assay. Distilled water was used to wash fixed cells, following which the cells were incubated for 10 min in a solution containing 2% Alizarin Red S, at a pH of 4.2 and indoor temperature. A Nikon camera was used to capture images of the histological samples. For quantifying alizarin Red S staining, 10% cetylpyridinium chloride was added into each well and the cells were incubated for 20 min to enable stain elution. Of the eluted stain, 100 μL was added into 96-well plates and a microplate reader was used to record the reading at 485 nm.

### Ovariectomized mouse model

Sham surgery or bilateral ovariectomies were conducted on female C57BL/6 mice of age ranging from 9–10 weeks, as described previously [42]. A week after ovariectomy, 30 mice were randomly assigned into 3 groups that included mice subject to sham operation (Sham), mice subjected to ovariectomy and vehicle treatment (OVX), and OVX mice subjected to paeoniflorin treatment (50 mg/kg). Ovariectomized mice were injected with either paeoniflorin or normal saline intragastrically on a daily basis. All mice were humanely euthanized with superfluous chloral hydrate 12 weeks following treatment, and their serum was collected. The hind limbs of the mice were dissected out and fixed in 4% paraformaldehyde.

For analyzing the osteolysis in mouse femoral condyles, a micro-CT scanner with high resolution (Skyscan 1176; Skyscan; Aartselaar, Belgium) was used (resolution of 9 mm, at 50 kV, 500 mA). For further quantitative analysis, a square area of interest (ROI) around the femoral condyle was selected following image reconstruction. The following analyses were performed: bone mineral content (BMC), bone mineral density (BMD), bone volume per tissue volume (BV/TV), trabecular thickness (Tb.th), trabecular number (Tb.N.), and trabecular separation (Tb.Sp.). A region from the growth plate measuring 1.4 mm (width), 0.9 mm (length), and 0.5 mm (thickness) was identified as the ROI. The fixed tibiae were decalcified with 12% EDTA and fixed in paraffin for haematoxylin and eosin (H&E) staining. ELISA kits (Westang, Shanghai, China) were used to measure the serum concentrations of RANKL and OPG, according to manufacturer instructions. Commercially available kits were used to measure serum bone formation and resorption markers: rat OC radioimmunoassay reagents (Biomedical Technologies, Stoughton, MA, USA) for measuring serum osteocalcin (OC) levels; ELISA systems (Immunodiagnostic Systems, Boldon, UK) for serum levels of markers of bone formation, N-terminal propeptide of type 1 procollagen (P1NP), and bone resorption, C-terminal cross-linked telopeptides of type I collagen (CTX-I).

### Biomechanical testing

A servohydraulic materials testing machine (MTS 858 Mini Bionix II; MTS Systems Corp., Minneapolis, MN, USA) was used to subject the femurs and vertebrae to three-point bending and axial compression. Prior to the testing, a preload with 0.5 N as the contact force was applied on the surface of the mid-shaft of femur at a speed range of 0.1–0.2 mm/min from the first stage to later stages. The lumbar vertebrae (L4) of rats were subjected to compression to failure at a displacement rate of 6 mm/min. TestStar II software was used to generate and analyze the load-deformation curves. Force and displacement data were measured and recorded following the fracture of the specimen. The load-deformation curve identified the following biomechanical parameters: maximum stress (MPa; the maximum load per cross-sectional area), maximum load (N; the load at the maximum failure point), Young’s modulus (GPa; maximum slope of the stress-strain curve). and stiffness (N/mm; the slope of the linear region).

### Statistical analysis

All results were expressed as mean ± standard deviation (SD). One-way ANOVA, followed by Dunnett post hoc test, was used to analyze the statistical differences between the different groups. Statistical significance was set at *P* < 0.05.
